# On a class of new means including the generalized Schwab-Borchardt mean

**DOI:** 10.1186/s13660-017-1412-1

**Published:** 2017-06-13

**Authors:** Mustapha Raïssouli, József Sándor

**Affiliations:** 1 Department of Mathematics, Science Faculty, Taibah University, P.O. Box 30097, Al Madinah Al Munawwarah, 41477 Saudi Arabia; 2 Department of Mathematics, Faculty of Science, Moulay Ismail University, Meknes, Morocco; 3 Department of Mathematics, Babes-Bolyai University, Str. Kogalniceanu nr. 1, Cluj-Napoca, 400084 Romania

**Keywords:** 26E60, bivariate mean, weighted mean, Schwab-Borchardt mean

## Abstract

The so-called Schwab-Borchardt mean plays an important role in the theory of (bivariate) means. It includes a lot of standard means, such as the logarithmic mean, the first and second Seiffert means and the Neuman-Sándor mean. In this paper, we investigate an approach which allows us to construct a class of new means. Such class includes the (generalized) Schwab-Borchardt mean and other old/new means derived as well.

## Introduction

Means arise in various contexts and contribute as good tool to solving many scientific problems. It has been proved, throughout a lot of works, that the theory of means is useful from the theoretical point of view as well as for practical purposes.

We understand by a mean a binary map between positive real numbers such that

1.1 $$ \forall a,b>0,\quad \min(a,b)\leq m(a,b)\leq\max(a,b). $$

Symmetric (resp. homogeneous, continuous) means are defined in the usual way. In the literature, an enormous amount of efforts has been devoted to understanding the theory of means in two variables when the involved means are symmetric in each variable. As far as we know, few papers were written about non-symmetric means. One of the interesting examples of non-symmetric mean is the so-called Schwab-Borchardt mean, denoted by *SB* and defined through [1, 2]

1.2 $$ \mathit{SB}:=\mathit{SB}(a,b)=\left \{ \textstyle\begin{array}{l@{\quad}l} \frac{\sqrt{b^{2}-a^{2}}}{\arccos(a/b)} & \mbox{if } 0< a< b,\\ \frac{\sqrt{a^{2}-b^{2}}}{\operatorname{arccosh}(a/b)} & \mbox{if } a>b, \end{array}\displaystyle \right . $$

with $\mathit{SB}(a,a)=a$. The importance of this non-symmetric mean lies in the fact that it includes a lot of symmetric means in the sense that

$$ L=\mathit{SB}(A,G),\qquad P=\mathit{SB}(G,A), \qquad T=\mathit{SB}(A,Q), \qquad M=\mathit{SB}(Q,A), $$

where

$$\begin{gathered} A:=A(a,b)=\frac{a+b}{2},\qquad G:=G(a,b)=\sqrt{ab}; \\L:=L(a,b)= \frac{a-b}{\ln a-\ln b}, \qquad L(a,a)=a; Q:=Q(a,b)=\sqrt{ \frac{a^{2}+b^{2}}{2}}; \\P:=P(a,b)= \frac{a-b}{4\arctan\sqrt{a/b}-\pi} = \frac{a-b}{2\arcsin\frac{a-b}{a+b}}= \frac {a-b}{4\arctan\frac{\sqrt{a}-\sqrt{b}}{\sqrt{a}+\sqrt{b}}},\qquad P(a,a)=a; \\T:=T(a,b)= \frac{a-b}{2\arctan\frac{a-b}{a+b}} = \frac{a-b}{2\arctan(a/b)-\pi/2}= \frac{a-b}{\arcsin\frac {a^{2}-b^{2}}{a^{2}+b^{2}}},\qquad T(a,a)=a; \\M:=M(a,b)= \frac{a-b}{2\arcsin h\frac{a-b}{a+b}},\qquad M(a,a)=a,\end{gathered} $$

are, respectively, known as the arithmetic mean, the geometric mean, the logarithmic mean, the quadratic mean, the first Seiffert mean [3], the second Seiffert mean [4] and the Neuman-Sándor mean [1]. For more details as regards recent developments for *SB*, see [1, 2, 5–14] for instance. The previous means satisfy the well-known chain of inequalities

1.3 $$ H< G< L< P< A< M< T< Q, $$

where the notation $m_{1}< m_{2}$, between two means $m_{1}$ and $m_{2}$, signifies that $m_{1}(a,b)< m_{2}(a,b)$ for all $a,b>0$ with $a\neq b$.

In [5, 6], Neuman introduced a generalization of *SB* itemized in the following. Let $p>1$ be a real number and set

1.4 $$ \mathit{SB}_{p}:=\mathit{SB}_{p}(a,b)=\left \{ \textstyle\begin{array}{l@{\quad}l} \frac{ (b^{p}-a^{p} )^{1/p}}{\arccos_{p}(a/b)} &\mbox{if } 0\leq a< b,\\ \frac{ (a^{p}-b^{p} )^{1/p}}{\arccos h_{p}(a/b)} & \mbox{if } a>b, \end{array}\displaystyle \right . $$

with $\mathit{SB}_{p}(a,a)=a$, where the notations $\arccos_{p}$ and $\operatorname{arccosh}_{p}$ refer to the *p*-generalized inverse of cosine and cosine-hyperbolic functions. The bivariate map $\mathit{SB}_{p}$ defines a non-symmetric homogeneous mean, the so-called *p*-Schwab-Borchardt mean, which when ${p=2}$ coincides with *SB*. A detailed study of the properties of $\mathit{SB}_{p}$ can be found in [5, 6]. As application, the following power means were derived there:

$$\begin{gathered} L_{p}=\mathit{SB}_{p} (A_{p/2},G ), \qquad P_{p}=\mathit{SB}_{p} (G,A_{p/2} ), \\ T_{p}=\mathit{SB}_{p} (A_{p/2}, A_{p} ), \qquad M_{p}=\mathit{SB}_{p} (A_{p},A_{p/2} ), \end{gathered} $$

where $A_{p}$ refers to the power (binomial) mean defined by $A_{p}:=A_{p}(a,b)= ((a^{p}+b^{p})/2 )^{1/p}$. The previous power means satisfy the chain of inequalities [5, 6]

1.5 $$ L_{p}< P_{p}< A_{p/2}< M_{p}< T_{p}< A_{p}. $$

An extension of $\mathit{SB}_{p}$ in a two-parameter mean (denoted by $\mathit{SB}_{p,q}$) can be found in [9].

## Weighted means

In this section, we state more notions needed later. We begin by the following definition.

### Definition 2.1

Let *m* be a symmetric mean. By weighted (or parameterized) *m*-mean, we understand a family $(m_{\lambda})_{0\leq\lambda\leq1}$ satisfying the following requirements:

(i) $m_{\lambda}$ is a mean, in the sense of (1.1), for all fixed $\lambda\in[0,1]$.
(ii) $m_{0}(a,b)=a$ and $m_{1}(a,b)=b$, for all $a,b>0$.
(iii) $m_{\lambda}(a,b)=m_{1-\lambda}(b,a)$, for all $a,b>0$ and every $\lambda\in[0,1]$.
(iv) $m_{\lambda}$ coincides with *m* if $\lambda=1/2$.

As standard examples of weighted means,

$$\begin{gathered} A_{\lambda}:=A_{\lambda}(a,b)=(1-\lambda)a+\lambda b,\qquad H_{\lambda }:=H_{\lambda}(a,b)= \bigl((1-\lambda) a^{-1}+ \lambda b^{-1} \bigr)^{-1}, \\ G_{\lambda}:=G_{\lambda}(a,b)=a^{1-\lambda}b^{\lambda},\qquad S_{\lambda}:=S_{\lambda}(a,b)= \bigl((1-\lambda)\sqrt{a}+\lambda\sqrt {b} \bigr)^{2}, \\ Q_{\lambda}:=Q_{\lambda}(a,b)=\sqrt{(1-\lambda)a^{2}+\lambda b^{2}}, \end{gathered}$$

are known in the literature as the weighted arithmetic, harmonic, geometric, square-root and root square (or quadratic) means, respectively. Such means satisfy

$$\min(a,b)\leq H_{\lambda}(a,b)\leq G_{\lambda}(a,b)\leq S_{\lambda }(a,b)\leq A_{\lambda}(a,b)\leq Q_{\lambda}(a,b)\leq \max(a,b) $$

for all $a,b>0$ and every $\lambda\in[0,1]$, with strict inequalities if and only if $a\neq b$ and $\lambda\in(0,1)$. These means are homogeneous/continuous, not symmetric unless $\lambda=1/2$ which corresponds to the simple means *A*, *H*, *G*, *S* and *Q*, respectively. The previous weighted means are included in a general class of means, so-called weighted power (binomial) mean, defined through

$$A_{r,\lambda}(a,b)= \bigl((1-\lambda)a^{r}+\lambda b^{r} \bigr)^{1/r}, $$

where $r\neq0$ is a parameter real number. Indeed, it is easy to see that

$$A_{1,\lambda}=A_{\lambda},\qquad A_{-1,\lambda}=H_{\lambda},\quad\quad A_{1/2,\lambda }=S_{\lambda},\qquad A_{2,\lambda}=Q_{\lambda},\qquad A_{0,\lambda}:=\lim_{r\rightarrow0}A_{r,\lambda}=G_{\lambda}. $$

A natural question arises from the above: what should be the reasonable weighted means associated to the symmetric means *L*, *T*, *M* and *P*. For the mean *L*, there are various weighted *L*-means that have been introduced in the literature; see [15–17] for instance. This understands that a given symmetric mean could have more one weighted mean. For the simplest means *A*, *H*, *G*, *S*, *Q* (and more generally $A_{r}$) only one weighted mean to each (as far as we know) is known in the literature. In fact, following two distinct points of view we can obtain different weighted means associated to the same symmetric mean, and this according to Definition 2.1 of course. As example, let us consider the symmetric logarithmic mean *L*. Its weighted mean is not simple to deduce from its explicit form in *a* and *b* previously mentioned. However, *L* has other equivalent forms known in the literature. In this paper (see Example 6.1), we will obtain via our approach a reasonable weighted *L*-means (*i.e.* satisfying all conditions of Definition 2.1). Another simple example explaining more the latter situation is stated in the following.

### Example 2.1

Let *C* be the contra-harmonic mean defined by $C(a,b)=\frac {a^{2}+b^{2}}{a+b}$. From this form we can immediately suggest a weighted *C*-mean defined as follows:

$$C_{\lambda}(a,b)=\frac{(1-\lambda)a^{2}+\lambda b^{2}}{(1-\lambda)a+\lambda b}, $$

which obviously satisfies all conditions of Definition 2.1. Now, it is easy to see that we can write *C* in the following equivalent form:

$$C(a,b)=\frac{a^{2}+b^{2}}{a+b}=a+b-\frac{2ab}{a+b}=2A(a,b)-H(a,b):=2A-H. $$

From the latter form of *C* we can suggest that a weighted *C*-mean is defined by $C_{\lambda}={2A_{\lambda}-H_{\lambda}}$ which also satisfies all conditions of Definition 2.1. Further, it is not hard to verify that the two previous *C*-weighted means are different. This justifies our claim.

Now, for the means *T*, *M* and *P*, related weighted means are hard to obtain from the explicit forms in *a* and *b* of these symmetric means. As we will see later, our approach investigated here leads us to introduce reasonable weighted means $T_{\lambda}$, $M_{\lambda}$ and $P_{\lambda}$ of *T*, *M* and *P*, respectively. To justify that our previous weighted means are good extensions of their related symmetric means, we establish that they satisfy the following chain of inequalities:

2.1 $$ H_{\lambda}< G_{\lambda}< L_{\lambda}< P_{\lambda}< A_{\lambda}< M_{\lambda }< T_{\lambda}< Q_{\lambda}. $$

It is worth mentioning that the two chains of inequalities (1.5) and (2.1) look alike while they are of course completely different.

## Basic notions and preliminary tools

Let us go back to the mean *SB*. It is easy to see that *SB*, defined by (1.2), can be written as follows:

$$\mathit{SB}(a,b)=\frac{\sqrt{|a^{2}-b^{2}|}}{ | \int_{1}^{a/b}\frac {dt}{\sqrt{|t^{2}-1|}} |}, $$

for all $a,b>0$, with $a\neq b$. In a more general point of view, the previous explicit form of *SB* may be included in the following form:

3.1 $$ (a,b)\longmapsto\frac{k(a,b)}{ \int_{1}^{a/b}f(t)\,dt}, $$

where *k* is a homogeneous bivariate map and *f* is a real function to be conveniently defined. We then ask the following question: under what conditions on *k* and *f* the binary map (3.1) defines a mean? In this section, we will discuss conditions about *k* while those of *f* are the purpose of the next section.

Let $k:(0,\infty)\times(0,\infty)\longrightarrow{\mathbb {R}}$ be a binary map satisfying the following requirements:

($c_{1}$)
*k* is homogeneous of degree 1, *i.e.* $k(\alpha a,\alpha b)=\alpha k(a,b)$ for all $a,b,\alpha>0$.
($c_{2}$)
$k(a,b)>0$ for $a>b$, $k(a,b)<0$ for $a< b$ and $k(a,a)=0$, for all $a,b>0$.
($c_{3}$)
The maps $t\longmapsto k(t,1)$ and $t\longmapsto t^{-1}k(t,1)$ are strictly monotone increasing on $(0,\infty)$.
($c_{4}$)
The map $t\longmapsto k(t,1)-t^{-1}k(t,1)$ is monotone increasing on $[1,\infty)$ and monotone decreasing on $(0,1]$.
($c_{5}$)
The map $t\longmapsto k(t,1)$ is continuously differentiable on $(0,1)\cup(1,\infty)$.

In what follows, we denote by ${\mathcal {K}}$ the set of all map $k:(0,\infty)\times(0,\infty)\longrightarrow{\mathbb {R}}$ satisfying the conditions ($c_{1}$)-($c_{5}$). It is not hard to see that ${\mathcal {K}}$ is a convex cone *i.e.*, for all $k,h\in{\mathcal {K}}$ and every $\alpha>0$ we have $h+k\in{\mathcal {K}}$ and $\alpha\cdot k\in{\mathcal {K}}$. The following definition may be stated.

### Definition 3.1

Let $k\in{\mathcal {K}}$ and define $k_{1},k_{2}:(0,\infty)\longrightarrow (0,\infty)$ as follows:

$$\begin{gathered} k_{1}(t):=\frac{d}{dt} \bigl(k(t,1) \bigr) \quad \mbox{for all } t>0, t\neq 1, \mbox{with } k_{1}(1)=1; \\k_{2}(t):=\frac{d}{dt} \bigl(t^{-1}k(t,1) \bigr)=- \frac{k(t,1)}{t^{2}}+\frac {1}{t}k_{1}(t) \quad\mbox{for all } t>0, t\neq1, \mbox{with } k_{2}(1)=1. \end{gathered}$$

The maps $k_{1}$ and $k_{2}$ will be called the components of *k* and we write $k=\langle k_{1},k_{2}\rangle$.

The next result, which summarizes the elementary properties of $k_{1}$ and $k_{2}$, will be needed later.

### Proposition 3.1

*With the above*, *the following assertions hold true*:

(i) $k_{1}$ *and* $k_{2}$ *are with positive values*, *both continuous on* $(0,1)\cup(1,\infty)$.
(ii) $k_{1}(t)>k_{2}(t)$ *if* $t>1$, $k_{1}(t)< k_{2}(t)$ *if* $0< t<1$.
(iii) *For all* $t>0$, *we have* $k_{1}(1/t)=t^{2}k_{2}(t)$ *and* $k_{2}(1/t)=t^{2}k_{1}(t)$.
(iv) *The map* $t\longmapsto k_{1}(t)/k_{2}(t)$ *is continuous on* $(0,\infty)$.

### Proof

(i) This follows from ($c_{3}$) and ($c_{5}$) while (ii) is a consequence of ($c_{4}$). The first relationship of (iii) can easily be proved by using the definition of $k_{1}$ with the homogeneity of *k* and the second one follows from the above by simple manipulation. Details are simple and therefore omitted here. For proving (iv), it is sufficient to show the continuity at $t=1$. Indeed, by the definition of $k_{1}$ and $k_{2}$ and a simple application of the reversed l’Hôpital rule we have

$$\lim_{t\rightarrow1}\frac{k_{1}}{k_{2}}(t)=\lim_{t\rightarrow1} \frac{\frac {d}{dt} (k(t,1) )}{ \frac{d}{dt} (t^{-1}k(t,1) )}=\lim_{t\rightarrow1}\frac {k(t,1)}{t^{-1}k(t,1)} =\lim _{t\rightarrow1}t=1=\frac{k_{1}}{k_{2}}(1). $$

The proof of the proposition is completed. □

For the sake of simplicity, we set $k_{12}=k_{1}/k_{2}$ throughout the following. Proposition 3.1(iv) asserts that the map $k_{12}:(0,\infty)\longrightarrow(0,\infty)$ is continuous on $(0,\infty )$.

An interesting example of $k\in{\mathcal {K}}$ is presented as follows. Let *q* be a real number such that $q>0$ and define the map *k* by

3.2 $$ k(a,b)=\left \{ \textstyle\begin{array}{l@{\quad}l} (a^{q}-b^{q} )^{1/q} & \mbox{if } a>b,\\ - (b^{q}-a^{q} )^{1/q} & \mbox{if } a< b,\\ 0 & \mbox{if } a=b. \end{array}\displaystyle \right . $$

In what follows, we denote by $q^{*}$ the conjugate of *q* defined by

$$\frac{1}{q}+\frac{1}{q^{*}}=1 \quad \textit{i.e. } q^{*}= \frac{q}{q-1}, $$

with the convention, if $q=1$ then $q^{*}=\infty$ and $1/q^{*}=0$. With this, the following lemma may be stated.

### Lemma 3.2

*Let* *k* *be given by* (3.2). *Then* $k\in{\mathcal {K}}$ *and its components are given by*

$$\begin{gathered} k_{1}(t)=\frac{t^{q-1}}{ (t^{q}-1 )^{1/q^{*}}} \quad \textit{if } t>1,\qquad k_{1}(t)=\frac{t^{q-1}}{ (1-t^{q} )^{1/q^{*}}} \quad\textit{if } 0< t< 1,\qquad k_{1}(1)=1; \\k_{2}(t)=\frac{1}{t^{2} (t^{q}-1 )^{1/q^{*}}} \quad \textit{if } t>1,\qquad k_{2}(t)=\frac{1}{t^{2} (1-t^{q} )^{1/q^{*}}} \quad \textit{if } 0< t< 1,\qquad k_{2}(1)=1. \end{gathered}$$

*Further*, *we have*

$$\forall t>0, \quad k_{12}(t):=\frac{k_{1}(t)}{k_{2}(t)}=t^{q+1}. $$

### Proof

The conditions ($c_{1}$), ($c_{2}$) and ($c_{5}$) are obviously satisfied while ($c_{3}$) and ($c_{4}$) follow after an elementary computation. Details are simple and therefore omitted here. □

We notice that $k_{1}$ and $k_{2}$ (of the previous lemma) are not continuous at $t=1$ unless $1/q^{*}=0$ *i.e.* $q=1$. This corresponds to the following simplest example.

### Example 3.1

Take $q=1$ in the previous lemma *i.e.* *k* is defined by $k(a,b)=a-b$ for all $a,b>0$. Simple computation leads to $k_{1}(t)=1$, $k_{2}(t)=1/t^{2}$ and $k_{12}(t)=t^{2}$, for every $t>0$.

Other examples may be stated as follows.

### Example 3.2

Let *k* be as follows: $k(a,b)=\sqrt{a^{2}-b^{2}}$ if $a\geq b$, $k(a,b)=-\sqrt{b^{2}-a^{2}}$ if $a\leq b$. This corresponds to (3.2) with $q=2$ and so we have

$$\begin{gathered} k_{1}(t)=\frac{t}{\sqrt{t^{2}-1}} \quad\mbox{if } t>1,\qquad k_{1}(t)=\frac {t}{\sqrt{1-t^{2}}} \quad\mbox{if } 0< t< 1,\qquad k_{1}(1)=1; \\ k_{2}(t)=\frac{1}{t^{2}\sqrt{t^{2}-1}} \quad\mbox{if } t>1,\qquad k_{2}(t)=\frac {1}{t^{2}\sqrt{1-t^{2}}} \quad\mbox{if } 0< t< 1,\qquad k_{2}(1)=1; \end{gathered}$$

and $k_{12}(t)=t^{3}$ for every $t>0$.

### Example 3.3

Taking $q=1/2$ and so $q^{*}=-1$, we obtain

$$\begin{gathered} k_{1}(t)=\frac{\sqrt{t}-1}{\sqrt{t}}\quad \mbox{if } t\geq1,\qquad k_{1}(t)=\frac{1-\sqrt{t}}{\sqrt{t}} \quad\mbox{if } 0< t\leq1,\qquad k_{1}(1)=1; \\ k_{2}(t)=\frac{\sqrt{t}-1}{t^{2}} \quad\mbox{if } t\geq1,\qquad k_{2}(t)=\frac {1-\sqrt{t}}{t^{2}} \quad\mbox{if } 0< t\leq1,\qquad k_{2}(1)=1; \end{gathered}$$

and $k_{12}(t)=t\sqrt{t}$ for all $t>0$.

The next lemma will also be needed in the sequel.

### Lemma 3.3

*Let* *k* *be defined by* (3.2) *and* *m* *be a homogeneous mean*. *Then we have*

3.3 $$ m \bigl(k_{1}(t),k_{2}(t) \bigr)=\left \{ \textstyle\begin{array}{l@{\quad}l} \frac{m (t^{q+1},1 )}{t^{2} (t^{q}-1 )^{1/q^{*}}} & \textit{if } t>1,\\ \frac{m (t^{q+1},1 )}{t^{2} (1-t^{q} )^{1/q^{*}}} & \textit{if } t< 1. \end{array}\displaystyle \right . $$

*Moreover*, *the map* $t\longmapsto m (k_{1}(t),k_{2}(t) )$, *defined from* $(0,\infty)$ *into itself*, *is discontinuous at* $t=1$, *unless* $q=1$.

### Proof

By the homogeneity of *m* we can write, for all $t>0$,

$$m \bigl(k_{1}(t),k_{2}(t) \bigr)=k_{2}(t)m \bigl(k_{12}(t),1 \bigr), $$

and so we obtain (3.3) by using the previous lemma. The remainder of the lemma can be checked in a simple way. □

## General approach

As already pointed out before, the previous section was devoted to listing convenient conditions on the binary map *k* in the aim that (3.1) defines a mean. In this section we will complete our previous discussion by stating favorable conditions about the function *f*.

Let $k\in{\mathcal {K}}$ and $k=\langle k_{1},k_{2}\rangle$ be as in the above section. Let $f:(0,\infty)\longrightarrow(0,\infty)$ be a function such that

4.1 $$ \forall t>0,\quad \min \bigl(k_{1}(t),k_{2}(t) \bigr)\leq f(t)\leq\max \bigl(k_{1}(t),k_{2}(t) \bigr). $$

By virtue of Definition 3.1, the maps $k_{1}$ and $k_{2}$ are locally integrable on $(0,\infty)$, *i.e.* integrable on every bounded subset of $(0,\infty)$. We therefore deduce from (4.1) that *f* is also locally integrable on $(0,\infty)$. Further, from (4.1) we deduce that $f(1)=1$ and with Proposition 3.1(4), the maps $t\longmapsto f(t)/k_{1}(t)$ and $t\longmapsto f(t)/k_{2}(t)$ are continuous on $(0,\infty )$ provided *f* is as well.

Now, we are in a position to state our first main result stated as follows.

### Theorem 4.1

*Let* $k\in{\mathcal {K}}$ *and* *f* *be satisfying the condition* (4.1) *and define the binary map* $M_{f,k}:(0,\infty)\times(0,\infty )\longrightarrow(0,\infty)$ *by*

4.2 $$ \bigl(M_{f,k}(a,b) \bigr)^{-1}= \frac{1}{k(a,b)} \int_{1}^{a/b}f(t)\,dt $$

*for all* $a,b>0$, *with* $M_{f,k}(a,a)=a$. *Then the following assertions hold*:

(i) $M_{f,k}$ *is a homogeneous mean*.
(ii) *If*, *moreover*, *f* *and* *k* *are such that*
  4.3 $$\begin{aligned}& \forall t>0,\quad f (1/t )=t^{2}f(t), \end{aligned}$$
  4.4 $$\begin{aligned}& \forall a,b>0,\quad k(a,b)=-k(b,a)\quad (\textit{i.e. } k \textit{ is antisymmetric}), \end{aligned}$$
  *then* $M_{f,k}$ *is symmetric*.
(iii) *If the function* *f* *is continuous then* $M_{f,k}$ *is also continuous*.

### Proof

(i) We first assume that $a>b$. According to Proposition 3.1 we have $k_{1}(t)\geq k_{2}(t)$ for all $1\leq t\leq a/b$. This, with (4.1), yields

4.5 $$ \begin{aligned}[b] \int_{1}^{a/b}k_{2}(t)&= \int_{1}^{a/b}\min \bigl(k_{1}(t),k_{2}(t) \bigr)\,dt \\ &\leq \int_{1}^{a/b}f(t)\,dt\leq \int_{1}^{a/b}\max \bigl(k_{1}(t),k_{2}(t) \bigr)\,dt= \int _{1}^{a/b}k_{1}(t)\,dt. \end{aligned}$$

Otherwise, by virtue of Definition 3.1 we can write

$$\begin{gathered} \int_{1}^{a/b}k_{1}(t)\,dt= \int_{1}^{a/b}\frac{d}{dt} \bigl(k(t,1) \bigr)\,dt=k(a/b,1)-k(1,1)=k(a/b,1)=\frac{k(a,b)}{b}, \\\int_{1}^{a/b}k_{2}(t)\,dt= \int_{1}^{a/b}\frac{d}{dt} \bigl(t^{-1}k(t,1) \bigr)\,dt=\frac{b}{a}k(a/b,1)=\frac{k(a,b)}{a}. \end{gathered}$$

Substituting these in (4.5), with the fact that $k(a,b)>0$ for $a>b$, we then obtain

4.6 $$ \frac{1}{a}\leq\frac{1}{k(a,b)} \int_{1}^{a/b}f(t)\,dt\leq\frac{1}{b}. $$

Now, if we assume that $a< b$ we can show in a similar manner

4.7 $$ \frac{1}{b}\leq\frac{1}{k(a,b)} \int_{1}^{a/b}f(t)\,dt\leq\frac{1}{a}. $$

Inequalities (4.6) and (4.7), with (4.2) and $M_{f,k}(a,a)=a$, show that

$$\min(a,b)\leq M_{f,k}(a,b)\leq\max(a,b) $$

for all $a,b>0$, *i.e.* $M_{f,k}$ is a mean. The fact that $M_{f,k}$ is homogeneous is immediate from (4.2).

(ii) Now, assume that (4.3) and (4.4) are satisfied. If we take $t=1/s$ as change of variables in (4.2) we then obtain

$$\bigl(M_{f,k}(a,b) \bigr)^{-1}=\frac{1}{k(a,b)} \int_{1}^{b/a}f(1/s) \biggl(\frac {-ds}{s^{2}} \biggr)=\frac{1}{k(b,a)} \int_{1}^{b/a}f(s)\,ds = \bigl(M_{f,k}(b,a) \bigr)^{-1}, $$

from which the symmetry for $M_{f,k}$ follows.

(iii) Assume that *f* is continuous. By virtue of the homogeneity of $M_{f,k}$, we need to prove that the map $x\longmapsto M_{f,k}(x,1)$ is continuous on $(0,\infty)$. By (4.2), it is clear that $x\longmapsto M_{f,k}(x,1)$ is continuous on $(0,1)\cup(1,\infty)$. Now, we have by (4.2) again and the l’Hôpital rule

$$\lim_{x\rightarrow1} \bigl(M_{f,k}(x,1) \bigr)^{-1}= \lim_{x\rightarrow 1}\frac{\int_{1}^{x}f(t)\,dt}{k(x,1)}=\lim_{x\rightarrow1} \frac{f(x)}{k_{1}(x)} =\frac{f(1)}{k_{1}(1)}=1, $$

since $x\longmapsto f(x)/k_{1}(x)$ is continuous on $(0,\infty)$. The continuity of $x\longmapsto M_{f,k}(x,1)$ follows. The proof of the theorem is complete. □

The reverse of Theorem 4.1(iii) is not always true, *i.e.* the mean $M_{f,k}$ could be continuous for not continuous *f*. The following example explains this situation.

### Example 4.1

Let $k\in{\mathcal {K}}$ be such that $k(a,b)=\sqrt{a^{2}-b^{2}}$ if $a\geq b$ and $k(a,b)=-\sqrt{b^{2}-a^{2}}$ if $0< a\leq b$. Let *f* be defined by

$$f(t)=\frac{1}{\sqrt{1-t^{2}}}\quad \mbox{if } 0< t< 1,\qquad f(t)=\frac {1}{\sqrt{t^{2}-1}}\quad \mbox{if } t>1,\qquad f(1)=1. $$

It is easy to see that such *f* satisfies (4.1) and its associated mean $M_{f,k}$ is exactly the Schwab-Borchardt mean *SB*. The mean *SB* is continuous while *f* is discontinuous at $t=1$. We will go back to this example later (see Example 5.1 and Section 5).

The following corollary is of great interest for practical purposes.

### Corollary 4.2

*Let* $k\in{\mathcal {K}}$, $k=\langle k_{1},k_{2}\rangle$ *and let* *m* *be a mean*. *Then the map* $m^{\sigma_{k}}$ *such that*

4.8 $$ \bigl(m^{\sigma_{k}}(a,b) \bigr)^{-1}= \frac{1}{k(a,b)} \int_{1}^{a/b}m \bigl(k_{1}(t),k_{2}(t) \bigr)\,dt $$

*for all* $a,b>0$, *with* $m^{\sigma_{k}}(a,a)=a$, *defines a homogeneous mean*. *Moreover*, *if* *k* *is antisymmetric and* *m* *is symmetric then* $m^{\sigma_{k}}$ *is also symmetric*.

### Proof

Let *m* be a mean and for $t>0$ we set $f(t)=m (k_{1}(t),k_{2}(t) )$ for some $k\in{\mathcal {K}}$. By (1.1), *f* satisfies (4.1) and by the previous theorem $m^{\sigma_{k}}$ is a homogeneous mean. If *m* is symmetric, Proposition 3.1(iv) implies that $f(1/t)=t^{2}f(t)$ for all $t>0$. The symmetry of $m^{\sigma_{k}}$ follows then from the previous theorem. □

### Remark 4.1

(i) The present approach extends that of [18] for a general class of maps *k* and for means not necessary homogeneous/symmetric/continuous. In fact, the above theorem and corollary give Theorem 2.1 and Corollary 2.2 of [18], respectively, when we consider the simplest *k* defined by $k(a,b)=a-b$ and *m* is a symmetric homogeneous continuous mean.

(ii) In what follows, the mean $m^{\sigma_{k}}$ defined by (4.8) will be called the $\sigma_{k}$-mean transform of *m*. For $k\in{\mathcal {K}}$ defined by (3.2), we write $m^{\sigma_{q}}$. In particular, $m^{\sigma_{2}}$ is that with *k* of Example 4.1. For $q=1$ in (3.2) *i.e.* $k(a,b)=a-b$, we simply write $m^{\sigma}$.

Choosing $k\in{\mathcal {K}}$ and *m* particular mean, we can obtain a lot of homogeneous (symmetric or not) means illustrating the above results. As trivial examples, it is not hard to check that $\min^{\sigma _{k}}=\max$ and $\max^{\sigma_{k}}=\min$, for all $k\in{\mathcal {K}}$. To the aim to not lengthen this section, we prefer to present other examples in another section below.

## Examples and properties of $m\longmapsto m^{\sigma_{k}}$

As a first example we present the following in form of result by virtue of its interest.

### Proposition 5.1

*Let* $k\in{\mathcal {K}}$. *Then the relationship* $A_{\lambda}^{\sigma _{k}}=H_{1-\lambda}$ *holds for all* $\lambda\in[0,1]$. *In particular* $A^{\sigma_{k}}=H$.

### Proof

Let $m=A_{\lambda}$ be the weighted arithmetic mean. For all $a,b>0$, $a\neq b$, we then have by (4.8)

$$\bigl(A_{\lambda}^{\sigma_{k}}(a,b) \bigr)^{-1}= \frac{1}{k(a,b)} \int _{1}^{a/b} \bigl((1-\lambda)k_{1}(t)+ \lambda k_{2}(t) \bigr)\,dt. $$

From the definition of $k_{1}$ and $k_{2}$, with a simple manipulation, it is easy to check that

$$\int_{1}^{a/b}k_{1}(t)\,dt=\frac{k(a,b)}{b} \quad \mbox{and}\quad \int _{1}^{a/b}k_{2}(t)\,dt= \frac{k(a,b)}{a}. $$

This immediately yields the desired result after a simple reduction. □

It is worth mentioning that in the previous relationship $A_{\lambda }^{\sigma_{k}}=H_{1-\lambda}$, $k\in{\mathcal {K}}$ is arbitrary. This could be coming from the fact that the weighted arithmetic mean $A_{\lambda}$ has a linear affine character.

Now we state more examples of interest.

### Example 5.1

Let *k* be as in Example 3.2 whose mean transform is denoted by $m^{\sigma_{2}}$.

(i) Let $m=G_{1/3}:=a^{2/3}b^{1/3}$. By Lemma 3.3, with an elementary computation, we have (here $q=2$ and so $q^{*}=2$)

$$\begin{gathered} G_{1/3} \bigl(k_{1}(t),k_{2}(t) \bigr)= \frac{G_{1/3}(t^{3},1)}{t^{2}\sqrt {t^{2}-1}}=\frac{1}{\sqrt{t^{2}-1}}\quad \mbox{if } t>1; \\ G_{1/3} \bigl(k_{1}(t),k_{2}(t) \bigr)= \frac{G_{1/3}(t^{3},1)}{t^{2}\sqrt{1-t^{2}}}=\frac {1}{\sqrt{1-t^{2}}}\quad\mbox{if } t< 1. \end{gathered}$$

By (4.8) we then deduce that $G_{1/3}^{\sigma_{2}}=\mathit{SB}$, that is, the $\sigma_{2}$-mean transform of $G_{1/3}$ is the Schwab-Borchardt mean *SB*.

(ii) Let $m=G_{2/3}:=a^{1/3}b^{2/3}$. By similar arguments as in the previous (i), we simply verify that $G_{2/3}^{\sigma_{2}}(a,b)=\mathit{SB}(b,a)$ *i.e.* $G_{2/3}^{\sigma_{2}}=\mathit{SB}^{T}$, where $\mathit{SB}^{T}$ denotes the mean transpose of *SB* defined by $\mathit{SB}^{T}(a,b)=\mathit{SB}(b,a)$ for all $a,b>0$.

We will go back again to this situation in section below.

### Example 5.2

Let $m=L$ be the logarithmic mean and let *k* be defined by (3.2). By the same tools as previously we have

$$\begin{gathered} L \bigl(k_{1}(t),k_{2}(t) \bigr)=\frac{L(t^{q+1},1)}{t^{2}(t^{q}-1)^{1/q^{*}}}= \frac {t^{q+1}-1}{(q+1)t^{2}(\ln t)(t^{q}-1)^{1/q^{*}}}\quad \mbox{if } t>1; \\ L \bigl(k_{1}(t),k_{2}(t) \bigr)=\frac{L(t^{q+1},1)}{t^{2}(1-t^{q})^{1/q^{*}}}= \frac {t^{q+1}-1}{(q+1)t^{2}(\ln t)(1-t^{q})^{1/q^{*}}}\quad \mbox{if } t< 1. \end{gathered}$$

By (4.8), with a simple manipulation and then with the change of variables $\ln t=u$, we obtain

$$\begin{gathered} \begin{aligned} \bigl(L^{\sigma_{q}}(a,b) \bigr)^{-1}&=\frac{1}{(q+1)(a^{q}-b^{q})^{1/q}} \int _{1}^{a/b}\frac{t^{q+1}-1}{t^{2}(\ln t)(t^{q}-1)^{1/q^{*}}}\,dt \\ &=\frac{1}{(q+1)(a^{q}-b^{q})^{1/q}} \int_{0}^{\ln(a/b)}\frac {e^{qt}-e^{-t}}{t(e^{qt}-1)^{1/q^{*}}}\,dt \quad\mbox{if } a>b;\end{aligned} \\\begin{aligned} \bigl(L^{\sigma_{q}}(a,b) \bigr)^{-1}&=\frac{1}{(q+1)(b^{q}-a^{q})^{1/q}} \int _{1}^{a/b}\frac{1-t^{q+1}}{t^{2}(\ln t)(t^{q}-1)^{1/q^{*}}}\,dt \\ &=\frac{1}{(q+1)(b^{q}-a^{q})^{1/q}} \int_{0}^{\ln(a/b)}\frac {e^{-t}-e^{qt}}{t(e^{qt}-1)^{1/q^{*}}}\,dt\quad \mbox{if } a< b. \end{aligned} \end{gathered} $$

If $q=1$ (and so $1/q^{*}=0$) the two previous formulas are reduced to the following:

$$\bigl(L^{\sigma}(a,b) \bigr)^{-1}(a,b)=\frac{1}{a-b} \int_{0}^{\ln(a/b)}\frac {\sinh(t)}{t}\,dt. $$

Further examples in a general context will be discussed in the next sections.

We now give some properties of the mean-map $m\longmapsto m^{\sigma _{k}}$. The first is stated as follows.

### Proposition 5.2

*Let* $k\in{\mathcal {K}}$ *be fixed*. *Then the two next statements hold*:

(i) *Let* $f,g:(0,\infty)\longrightarrow(0,\infty)$ *be two functions satisfying* (4.1) *with* $f\leq g$. *Then* $M_{f,k}(a,b)\geq M_{g,k}(a,b)$ *for all* $a,b>0$.
(ii) *Let* $m_{1}$ *and* $m_{2}$ *be two means such that* $m_{1}< m_{2}$. *Then* $m_{1}^{\sigma_{k}}>m_{2}^{\sigma_{k}}$.

### Proof

This follows from (4.2) and (4.8), respectively. Details are simple and therefore omitted here. □

When we have to compare two means $m_{1}$ and $m_{2}$ which are homogeneous but not symmetric, we usually have inequalities in the form

$$m_{1}(a,b)< m_{2}(a,b)\quad \mbox{if } a< b;\qquad m_{1}(a,b)>m_{2}(a,b) \quad\mbox{if } a>b. $$

For example, it is easy to see that if $\lambda<\mu$ then $A_{\lambda }(a,b)< A_{\mu}(a,b)$ whenever $a< b$. It will then be interesting to see that if the previous proposition can be improved in this sense. The answer is positive as confirmed by the following result.

### Proposition 5.3

*Let* $m_{1}$ *and* $m_{2}$ *be two means such that*

$$m_{1}(a,b)< m_{2}(a,b) \quad\textit{if } a< b;\qquad m_{1}(a,b)>m_{2}(a,b) \quad\textit{if } a>b. $$

*Then we have*

$$m_{1}^{k}(a,b)>m_{2}^{k}(a,b)\quad \textit{if } a< b;\qquad m_{1}^{k}(a,b)< m_{2}^{k}(a,b) \quad \textit{if } a>b $$

*for each* $k\in{\mathcal {K}}$.

### Proof

First, we recall that $k(a,b)>0$ for $a>b$ and $k(a,b)<0$ if $a< b$. Secondly, we have $k_{1}(t)>k_{2}(t)$ for $t>1$ and $k_{1}(t)< k_{2}(t)$ if $0< t<1$ (see Proposition 3.1). This, with a simple manipulation on (4.8), yields the desired result. Details are simple and therefore omitted here. □

The following example illustrates the previous proposition.

### Example 5.3

It is easy to see that $G_{1/3}(a,b)< G_{2/3}(a,b)$ for all $a< b$ (and so $G_{1/3}(a,b)>G_{2/3}(a,b)$ if $a>b$, since $G_{\lambda }(a,b)=G_{1-\lambda}(b,a)$). By Proposition 5.3, with Example 5.1, we then deduce

$$\mathit{SB}(a,b)>\mathit{SB}^{T}(a,b)=\mathit{SB}(b,a)\quad \mbox{if } a< b, $$

which is a well-known result; see [1].

Now, we can observe the next question: let $m_{1}$ and $m_{2}$ be two means and $k,h\in{\mathcal {K}}$ such that $m_{1}^{\sigma_{k}}=m_{2}^{\sigma_{h}}$. We ask if this implies that $m_{1}=m_{2}$ and $k=h$. Proposition 5.1 shows that it is not true, since $A_{\lambda}^{\sigma_{k}}=H_{1-\lambda}=A_{\lambda}^{\sigma_{h}}$ for all $k,h\in{\mathcal {K}}$. However, the next result may be stated.

### Proposition 5.4

*Let* $m_{1}$ *and* $m_{2}$ *be two continuous homogeneous means such that* ${m_{1}^{\sigma_{k}}=m_{2}^{\sigma_{k}}}$ *for some* $k\in{\mathcal {K}}$. *Assume that the map* $k_{12}:(0,\infty)\longrightarrow(0,\infty)$ *is onto*. *Then we have* $m_{1}=m_{2}$.

### Proof

If $m_{1}^{\sigma_{k}}=m_{2}^{\sigma_{k}}$ for the same $k\in{\mathcal {K}}$ then (4.8) yields (by setting $x=b/a$)

$$ \forall x>0,\quad \int_{1}^{x}m_{1} \bigl(k_{1}(t),k_{2}(t) \bigr)\,dt= \int _{1}^{x}m_{2} \bigl(k_{1}(t),k_{2}(t) \bigr)\,dt. $$

We then deduce

$$m_{1} \bigl(k_{1}(t),k_{2}(t) \bigr)=m_{2} \bigl(k_{1}(t),k_{2}(t) \bigr), $$

or by the homogeneity of $m_{1}$ and $m_{2}$,

$$m_{1} \bigl(k_{12}(t),1 \bigr)=m_{2} \bigl(k_{12}(t),1 \bigr) $$

almost everywhere for $t>0$. Let $a,b>0$. Since $k_{12}$ is onto, there exists $t>0$ such that $k_{12}(t)=a/b$. We then deduce $m_{1}(a/b,1)=m_{2}(a/b,1)$, or by the homogeneity of $m_{1}$ and $m_{2}$ again, $m_{1}(a,b)=m_{2}(a,b)$, almost everywhere for $a,b>0$. Since $m_{1}$ and $m_{2}$ are continuous we therefore infer that $m_{1}(a,b)=m_{2}(a,b)$ for all $a,b>0$, so completing the proof. □

### Example 5.4

Let *k* be defined as in (3.2). Then $k_{12}(t)=t^{q+1}$ which is onto for $q>0$. It follows that if $m_{1}$ and $m_{2}$ are as in the previous proposition, with $m_{1}^{\sigma_{q}}=m_{2}^{\sigma_{q}}$ for some $q>0$ then $m_{1}=m_{2}$.

## Application 1: power mean including *SB*

As already pointed out before, this section displays various applications of the above theoretical approach for constructing some new power means including, among other, the Schwab-Borchardt mean. The next result, giving us a lot of power homogeneous means, is of great interest.

### Theorem 6.1

*Let* *q*, *λ* *be two real numbers such that* $q>0$ *and* $0\leq\lambda \leq1$. *Then the binary map* $X_{q,\lambda}$ *defined by* $X_{q,\lambda }(a,a)=a$ *and*

6.1 $$ \bigl(X_{q,\lambda}(a,b) \bigr)^{-1}=\left \{ \textstyle\begin{array}{l@{\quad}l} {\frac{1}{ (a^{q}-b^{q} )^{1/q}}\int_{1}^{a/b}\frac {t^{q-1-\lambda(q+1)}}{ (t^{q}-1 )^{1/q^{*}}}\,dt}& \textit{if } a>b,\\ {\frac{-1}{ (b^{q}-a^{q} )^{1/q}}\int_{1}^{a/b}\frac {t^{q-1-\lambda(q+1)}}{ (1-t^{q} )^{1/q^{*}}}\,dt} &\textit{if } a< b, \end{array}\displaystyle \right . $$

*is a homogeneous mean*, *symmetric provided* $\lambda=1/2$.

### Proof

Let *k* be as in (3.2) and take $m=G_{\lambda}:=a^{1-\lambda }b^{\lambda}$ the weighted geometric mean. Corollary 4.2 asserts that $X_{q,\lambda}$ defined by $X_{q,\lambda}(a,a)=a$ and

6.2 $$ \bigl(X_{q,\lambda}(a,b) \bigr)^{-1}= \frac{1}{k(a,b)} \int_{1}^{a/b} \bigl(k_{1}(t) \bigr)^{1-\lambda} \bigl(k_{2}(t) \bigr)^{\lambda}\,dt $$

for all $a,b>0$, $a\neq b$, is a homogeneous mean, symmetric if $G_{\lambda}$ is also symmetric *i.e.* ${\lambda=1/2}$. Replacing $k_{1}$ and $k_{2}$ by their explicit expressions given by Lemma 3.2, (6.2) yields the desired result after a simple computation. □

Now, let us present the following example of interest.

### Example 6.1

If in the previous theorem we take $q=1$ (and so $q^{*}=\infty$, $1/q^{*}=0$), it is easy to see that, for all $a,b>0$, $a\neq b$, we have

$$\bigl(X_{1,\lambda}(a,b) \bigr)^{-1}=\frac{1}{a-b} \int_{1}^{a/b}t^{-2\lambda}\,dt, $$

which after simple computation leads to

6.3 $$ X_{1,\lambda}(a,b)=\frac{(1-2\lambda)(a-b)}{ (a/b )^{1-2\lambda}-1}. $$

Moreover, $X_{1,1/2}$ is symmetric and it is not hard to verify that

$$X_{1,1/2}(a,b):=\lim_{\lambda\rightarrow1/2}X_{1,\lambda}(a,b)= \frac{a-b}{\ln a-\ln b}=L(a,b). $$

From (6.3) we immediately obtain $X_{1,0}(a,b)=b$ and $X_{1,1}(a,b)=a$. Further, a simple verification asserts that $X_{1,\lambda}(a,b)=X_{1,1-\lambda}(b,a)$ for all $a,b>0$ and each $\lambda\in[0,1]$. These, with the fact that $A_{\lambda}^{\sigma }=H_{1-\lambda}$, allow us to set $X_{1,\lambda}=L_{1-\lambda}$, *i.e.* with (6.3)

$$L_{\lambda}(a,b)=\frac{(2\lambda-1)(a-b)}{(a/b)^{2\lambda-1}-1},\quad\quad L_{\lambda}(a,a)=a, $$

as weighted logarithmic mean, according to Definition 2.1. This weighted logarithmic mean is simpler than those introduced in [16] and [17]. Another *L*-weighted mean will be introduced by analogy with those of *T* and *M*. See Section 10 below.

Now, taking $q=2$ in the above theorem we obtain the following corollary.

### Corollary 6.2

*Let* *λ* *be such that* $0\leq\lambda\leq1$. *Then the binary map* $X_{2,\lambda}$ *defined by* $X_{2,\lambda}(a,a)=a$ *and*

6.4 $$ \bigl(X_{2,\lambda}(a,b) \bigr)^{-1}=\left \{ \textstyle\begin{array}{l@{\quad}l} {\frac{1}{\sqrt{a^{2}-b^{2}}}\int_{0}^{\operatorname{argch}(a/b)} (\operatorname{ch}t )^{1-3\lambda}\,dt}& \textit{if } a>b,\\ {\frac{1}{\sqrt{b^{2}-a^{2}}}\int_{0}^{\arccos(a/b)} (\cos t )^{1-3\lambda}\,dt} &\textit{if } a< b , \end{array}\displaystyle \right . $$

*is a homogeneous mean*, *symmetric for* $\lambda=1/2$.

### Proof

If $q=2$ then $q^{*}=2$. Making the change of variables $s=\operatorname{argch} t$ and $s=\arccos t$ in the two integrals of (6.1), respectively, we obtain the desired result after an elementary manipulation. Details are simple and therefore omitted here. □

Now, choosing particular values for *q*, *λ* in the above, we can state the following interesting examples.

### Example 6.2

(i) Taking $\lambda=1/3\in[0,1]$ in (6.4), we find (after a simple computation) the Schwab-Borchardt mean *i.e.* $X_{2,1/3}=\mathit{SB}$. Since $G_{\lambda}(a,b)=G_{1-\lambda}(b,a)$, we have $X_{q,\lambda}(a,b)=X_{q,1-\lambda}(b,a)$ for fixed $q>0$. It follows that $X_{2,2/3}(a,b)=\mathit{SB}(b,a)$.

(ii) Theorem 6.1, with Remark 4.1, can be formulated as follows: For all $\lambda\in[0,1]$ and $q>0$, we have $G_{\lambda }^{\sigma_{q}}=X_{q,\lambda}$. In particular, Example 6.1 can be formulated as $G_{\lambda}^{\sigma}=L_{1-\lambda}$. Also, the previous (i) means that $G_{1/3}^{\sigma_{2}}=\mathit{SB}$ and $G_{2/3}^{\sigma_{2}}=\mathit{SB}^{T}$.

Now, let us observe another interesting special situation given in the following example.

### Example 6.3

Assume that here $q>1$. If in (6.1) we take $\lambda=\frac {q-1}{q+1}\in(0,1)$, then we obtain (in a brief form for the sake of simplicity)

$$G_{\frac{q-1}{q+1}}^{\sigma_{q}}(a,b) =\frac{1}{|a^{q}-b^{q}|^{1/q}}\bigg| \int_{1}^{a/b}\frac {dt}{|t^{q}-1|^{1/q^{*}}}\bigg| $$

for all $a,b>0$ with $a\neq b$. This generalized mean is to compare with the so-called *q*-Schwab-Borchardt mean $\mathit{SB}_{q}$, introduced and studied in [5, 6].

### Example 6.4

Corollary 6.2 asserts that $X_{2,1/2}$ is a (homogeneous) symmetric mean. We can then ask what is the explicit form of this mean. If we set

6.5 $$ \alpha(x)= { \int_{1}^{x}\frac{dt}{\sqrt{t(t^{2}-1)}}}\quad\mbox{if } x>1,\qquad \alpha(x)={ \int_{x}^{1}\frac{dt}{\sqrt {t(1-t^{2})}}}\quad\mbox{if } x< 1, $$

then we can easily see that

$$ X_{2,1/2}(a,b)= \frac{\sqrt{a^{2}-b^{2}}}{\alpha (a/b )}\quad\mbox{if } a>b,\qquad X_{1/2,2}(a,b)=\frac{\sqrt{b^{2}-a^{2}}}{\alpha (a/b )}\quad\mbox{if } a< b, $$

with $X_{2,1/2}(a,a)=a$. By the simple change of variables $t=1/s$ in the integrals of (6.5) we can verify that $\alpha(a/b)=\alpha (b/a)$ for all $a,b>0$. Summarizing, $X_{2,1/2}$ is a homogeneous symmetric mean defined through

6.6 $$ \forall a,b>0, a\neq b,\quad X_{2,1/2}(a,b)= \frac{\sqrt {|a^{2}-b^{2}|}}{\alpha(a/b)}, \quad\mbox{with } X_{2,1/2}(a,a)=a, $$

where $\alpha:(0,\infty)\longrightarrow(0,\infty)$ is defined by

$$\forall x>0,\quad \alpha(x)= \biggl\vert \int_{1}^{x}\frac{dt}{\sqrt {t|t^{2}-1|}} \biggr\vert . $$

We can then see $X_{2,\lambda}$ defined by (6.4) as weighted mean associated to the symmetric mean $X_{2,1/2}$ given through (6.6). It seems that explicit computation of $\alpha(x)$ and so that of $X_{2,1/2}(a,b)$, for all $a,b>0$, in terms of elementary functions is impossible.

## Application 2: weighted means of *T* and *M*

In this section we give more application of our present approach. In particular, weighted mean associated to the symmetric means *T* will be investigated. We preserve the same notations as previously and we start with the following central result.

### Theorem 7.1

*Let* *q*, *λ* *be two real numbers such that* $q>0$ *and* $0\leq\lambda \leq1$. *Then the binary map* $Y_{q,\lambda}$ *defined by* $Y_{q,\lambda }(a,a)=a$ *and*

$$ \bigl(Y_{q,\lambda}(a,b) \bigr)^{-1}=\left \{ \textstyle\begin{array}{l@{\quad}l} {\frac{1}{ (a^{q}-b^{q} )^{1/q}}\int_{1}^{a/b}\frac{t^{q-1}\,dt}{ (1-\lambda+\lambda t^{q+1} ) (t^{q}-1 )^{1/q^{*}}}} &\textit{if } a>b,\\ {\frac{-1}{ (b^{q}-a^{q} )^{1/q}}\int_{1}^{a/b}\frac{t^{q-1}\,dt}{ (1-\lambda+\lambda t^{q+1} ) (1-t^{q} )^{1/q^{*}}}} &\textit{if } a< b, \end{array}\displaystyle \right . $$

*is a homogeneous continuous mean*, *symmetric if* $\lambda=1/2$.

### Proof

Let $k\in{\mathcal {K}}$ be defined by (3.2) and

$$m(a,b)=H_{\lambda}(a,b):=\frac{ab}{\lambda a+(1-\lambda)b} $$

be the weighted harmonic mean. Corollary 4.2 with Lemma 3.3 yields the desired result after a simple computation. Details are similar to the proof of Theorem 6.1. □

Generally, $Y_{q,\lambda}$ previously introduced cannot be explicitly computed, except for few particular values of *q*, such as $q=1$ and $q=1/2$. The case $q=1$, which corresponds to $k(a,b)=a-b$, is presented in the following corollary.

### Corollary 7.2

*The binary map* $Y_{1,\lambda}$ *defined by*

$$Y_{1,\lambda}(a,b)=\frac{\sqrt{\lambda(1-\lambda)}(a-b)}{ \arctan (\sqrt{\lambda/(1-\lambda)}(a/b) )-\arctan\sqrt{\lambda /(1-\lambda)}}, $$

*with* $Y_{1,\lambda}(a,a)=a$, *defines a homogeneous continuous mean*, *symmetric if* $\lambda=1/2$.

### Proof

Taking $q=1$ (and so $1/q^{*}=0$) in Theorem 7.1 we obtain

$$\bigl(Y_{1,\lambda}(a,b) \bigr)^{-1}=\frac{1}{a-b} \int_{1}^{a/b}\frac {dt}{1-\lambda+\lambda t^{2}}, $$

which after an elementary computation (by change of variables) yields the desired result. □

Now, we will analyze the above mean $Y_{1,\lambda}$ in the aim to obtain convenient weighted means of *T*, *M* and *P*. First, it is easy to see that, for all $a,b>0$ with $a\neq b$, one has

$$Y_{1,1/2}(a,b)=\frac{a-b}{2\arctan(a/b)-\pi/2}, $$

*i.e.* $Y_{1,1/2}$ is nothing other than the second Seiffert mean *T*. It is also easy to check that the relationship $Y_{1,\lambda }(a,b)=Y_{1,1-\lambda}(b,a)$ holds for all $a,b>0$ and every $\lambda\in [0,1]$. Further, it is not hard to verify that $Y_{1,0}(a,b)=b$ and $Y_{1,1}(a,b)=a$. As for $L_{\lambda}$, this with Definition 2.1 allows us to define the weighted *T*-mean as follows: $T_{\lambda}=Y_{1,1-\lambda}$ *i.e.*

7.1 $$ T_{\lambda}:=T_{\lambda}(a,b)=\frac{\sqrt{\lambda(1-\lambda)}(a-b)}{ \arctan (\sqrt{(1-\lambda)/\lambda}(a/b) )-\arctan\sqrt {(1-\lambda)/\lambda}} $$

for all $a,b>0$, with $T_{\lambda}(a,a)=a$. Using the equality

$$\arctan x -\arctan y=\arctan\frac{x-y}{1+xy}, $$

valid for all $x,y>0$, it is easy to see that $T_{\lambda}$ given by (7.1) can be written as follows:

7.2 $$ T_{\lambda}(a,b)=\frac{\sqrt{\lambda(1-\lambda)}(a-b)}{ \arctan\frac{\sqrt{\lambda(1-\lambda)}(a-b)}{A_{\lambda}(a,b)}},\qquad T_{\lambda}(a,a)=a. $$

After obtaining $T_{\lambda}$ from our previous approach, we can now derive the *M*-weighted mean by a simple observation over (7.2) together with a comparison between the explicit forms of *T* and *M*. We can then automatically suggest that

7.3 $$ M_{\lambda}(a,b)=\frac{\sqrt{\lambda(1-\lambda)}(a-b)}{ \operatorname{arcsinh}\frac{\sqrt{\lambda(1-\lambda)}(a-b)}{A_{\lambda}(a,b)}},\qquad M_{\lambda}(a,a)=a, $$

is a weighted mean associated to *M*. Of course, $M_{\lambda}$ should satisfy all conditions of Definition 2.1, which can easily be checked.

It is suitable to give more justification for our above weighted means. The following result is another reason of such suggestion.

### Proposition 7.3

*For all* $\lambda\in[0,1]$ *we have*

$$T_{\lambda}=\mathit{SB}(A_{\lambda},Q_{\lambda}),\qquad M_{\lambda}=\mathit{SB}(Q_{\lambda },A_{\lambda}). $$

### Proof

By virtue of the explicit forms (7.2) and (7.3) we can assume, without loss of generality, $a>b$. It is easy to see that

$$Q_{\lambda}^{2}-A_{\lambda}^{2}=\lambda(1-\lambda) (a-b)^{2}. $$

Further,

$$\arccos\frac{A_{\lambda}}{Q_{\lambda}}=\arccos\frac{(1-\lambda)x+\lambda}{ \sqrt{(1-\lambda)x^{2}+\lambda}}:=\Phi(x) $$

and

$$\operatorname{arccosh}\frac{Q_{\lambda}}{A_{\lambda}}=\operatorname{arccosh}\frac {\sqrt{(1-\lambda)x^{2}+\lambda}}{(1-\lambda)x+\lambda}:=\Psi(x), $$

where we set $x=a/b$. Simple computation leads to (after simplification and reduction)

$$\Phi{'}(x)=\frac{\sqrt{\lambda(1-\lambda)}}{(1-\lambda)x^{2}+\lambda}, \qquad \Psi{'}(x)= \frac{\sqrt{\lambda(1-\lambda)}}{ ((1-\lambda )x+\lambda ) \sqrt{(1-\lambda)x^{2}+\lambda}}, $$

and by simple integration (since $\Phi(1)=\Psi(1)=0$) we find

$$\Phi(x)=\arctan\frac{\sqrt{\lambda(1-\lambda)}(x-1)}{(1-\lambda )x+\lambda},\qquad \Psi(x)=\operatorname{arcsinh}\frac{\sqrt{\lambda(1-\lambda )}(x-1)}{(1-\lambda)x+\lambda}. $$

This, with $x=a/b$, yields

$$\arccos\frac{A_{\lambda}}{Q_{\lambda}}= \arctan\frac{\sqrt{\lambda(1-\lambda)}(a-b)}{(1-\lambda)a+\lambda b} $$

and

$$\operatorname{arccosh}\frac{Q_{\lambda}}{A_{\lambda}} =\operatorname{arcsinh}\frac{\sqrt{\lambda(1-\lambda)}(a-b)}{(1-\lambda)a+\lambda b}. $$

The two desired equalities are so obtained. □

We can give more results justifying that the previous $T_{\lambda}$ and $M_{\lambda}$ are really reasonable weighted means associated to *T* and *M*, respectively. In fact, the chain of inequalities

7.4 $$ (H_{\lambda}< )\,G_{\lambda}< L_{\lambda}< A_{\lambda}< M_{\lambda}< T_{\lambda }< Q_{\lambda} $$

holds for every $\lambda\in(0,1)$. Indeed, since all involved means here are homogeneous, we can show this chain of inequalities by comparing the associated functions of these means. Such method is classical and very known. We omit all details here, because we will show again this chain in another way. See the next section.

Now, about the weighted mean of *P*. This needs a long discussion which will be developed in Section 10 below. Other *L*-weighted means will be discussed there, by analogy with those of *T* and *M* previously investigated.

## Generated function

Let $k\in{\mathcal {K}}$ with $k=\langle k_{1},k_{2}\rangle$ and *m* be a homogeneous mean. We start this section by stating the following definition.

### Definition 8.1

Assume that the map $x\longmapsto\frac{k(x,1)}{m(x,1)}$ is continuously differentiable on $(0,1)\cup(1,\infty)$. We then set

8.1 $$ \forall x>0,x\neq1,\quad F_{m}^{k}(x)= \frac{d}{dx} \biggl(\frac {k(x,1)}{m(x,1)} \biggr),\qquad F_{m}^{k}(1)=1. $$

If, moreover, $F_{m}^{k}$ satisfies (4.1) then $F_{m}^{k}$ is called the *k*-generated function of the mean *m*. If *k* is such that $k(a,b)=a-b$ we simply write $F_{m}$ (the generated function of *m*).

Since $x\longmapsto k(x,1)$ is continuously differentiable on $(0,1)\cup (1,\infty)$, so is $x\longmapsto F_{m}^{k}(x)$ provided that $x\longmapsto m(x,1)$ is as well.

### Example 8.1

Let *k* be as $k(a,b)=a-b$. Simple computations lead to, for all $x>0$,

$$F_{A_{\lambda}}(x)=\frac{1}{ ((1-\lambda)x+\lambda )^{2}},\qquad F_{H_{\lambda}}(x) = \frac{1-\lambda+\lambda x^{2}}{x^{2}},\qquad F_{G_{\lambda}}(x)=x^{\lambda -2} (1-\lambda+\lambda x ). $$

For the *Q*-weighted mean we have

$$F_{Q_{\lambda}}(x)=\frac{(1-\lambda)x+\lambda}{((1-\lambda)x^{2}+\lambda) \sqrt{(1-\lambda)x^{2}+\lambda}}, $$

while for the weighted logarithmic mean $L_{\lambda}$ introduced in Example 6.1 we easily verify that

$$F_{L_{\lambda}}(x)=x^{2(\lambda-1)}. $$

### Example 8.2

Let *k* be as in the previous example and consider the weighted mean $T_{\lambda}$ defined by (7.2). We have

$$F_{T_{\lambda}}(x)=\frac{1}{\sqrt{\lambda(1-\lambda)}} \frac{d}{dx}\arctan \frac{\sqrt{\lambda(1-\lambda)}(x-1)}{(1-\lambda )x+\lambda}, $$

which after elementary computation of the derivative yields

$$F_{T_{\lambda}}(x)=\frac{1}{(1-\lambda)x^{2}+\lambda}. $$

For $M_{\lambda}$, computation similar to $T_{\lambda}$ leads to

$$F_{M_{\lambda}}(x)=\frac{1}{ ((1-\lambda)x+\lambda )\sqrt {(1-\lambda)x^{2}+\lambda}}. $$

### Example 8.3

Let *k* be defined by $k(a,b)=\sqrt{a^{2}-b^{2}}$ if $a\geq b$ and $k(a,b)=-\sqrt{b^{2}-a^{2}}$ if $a\leq b$.

(i) According to (1.2) with (8.1), it is easy to see that

$$\forall x>0,\quad F_{\mathit{SB}}^{k}(x)= \frac{1}{\sqrt{|x^{2}-1|}},\quad \mbox{with } F_{\mathit{SB}}^{k}(1)=1. $$

(ii) By similar arguments, we obtain (after elementary computations)

$$F_{A_{\lambda}}^{k}(x)=\frac{ (\lambda x+1-\lambda )}{ ((1-\lambda)x+\lambda )^{2}\sqrt{|x^{2}-1|}},\qquad F_{A_{\lambda}}^{k}(1)=1 $$

and

$$F_{G_{\lambda}}^{k}(x)=\frac{x^{\lambda-2} (\lambda x^{2}+1-\lambda )}{\sqrt{|x^{2}-1|}},\qquad F_{G_{\lambda}}^{k}(1)=1. $$

In particular,

$$F_{A}^{k}(x)=\frac{2}{(x+1)\sqrt{|x^{2}-1|}},\qquad F_{A}^{k}(1)=1 $$

and

$$F_{G}^{k}(x)=\frac{x^{2}+1}{2x\sqrt{x}\sqrt{|x^{2}-1|}},\qquad F_{G}^{k}(1)=1. $$

We left to the reader the task for computing $F_{H_{\lambda}}^{k}$ in a similar manner.

Now we state the following result.

### Proposition 8.1

*Let* $k\in{\mathcal {K}}$ *and* *m* *be a homogeneous mean*. *Let* $F_{m}^{k}$ *be the* *k*-*generated function of* *m*. *Then we have*

8.2 $$ \forall a,b>0,\quad \bigl(m(a,b) \bigr)^{-1}= \frac{1}{k(a,b)} \int _{1}^{a/b}F_{m}^{k}(t)\,dt. $$

### Proof

It is a simple exercise whose details are omitted here. □

In order to state an application of the above, we introduce more notation. If $f,g:(0,\infty)\longrightarrow(0,\infty)$ are such that $f(x)< g(x)$ for all $x>0$ with $x\neq1$ then we write $f\prec g$. With this, we have the following.

### Proposition 8.2

*Let* $m_{1}$, $m_{2}$ *be two homogeneous means*. *Then the following assertions hold*:

(i) *If* $F_{m_{1}}^{k}=F_{m_{2}}^{k}$ *for some* $k\in{\mathcal {K}}$ *then we have* $m_{1}=m_{2}$.
(ii) *If* $F_{m_{1}}^{k}\prec F_{m_{2}}^{k}$ *for some* $k\in{\mathcal {K}}$ *then* $m_{1}>m_{2}$.

### Proof

This follows immediately from (8.2). Details are simple. □

The assertion (i) of the previous proposition means that the map $m\longmapsto F_{m}^{k}$, for fixed $k\in{\mathcal {K}}$, is one-to-one (on the set of homogeneous means). It is also possible to show that the map $k\longmapsto F_{m}^{k}$, for fixed homogeneous mean *m*, is one-to-one. Assertion (ii) is more interesting and can be used for showing some mean inequalities. In particular, the chain of inequalities (7.4) can be proved here in a simple and fast way as explained in the following.

### Theorem 8.3

*For all* $\lambda\in(0,1)$ *we have*

8.3 $$ (H_{\lambda}< )\, G_{\lambda}< L_{\lambda}< A_{\lambda}< M_{\lambda }< T_{\lambda}< Q_{\lambda}. $$

### Proof

We show $G_{\lambda}< L_{\lambda}< A_{\lambda}$. By Proposition 8.2, with Example 8.1, it is sufficient to prove that, for all $x>0$ with $x\neq1$,

$$\frac{1}{ ((1-\lambda)x+\lambda )^{2}}< x^{2(\lambda-1)}< x^{\lambda -2} (1-\lambda+\lambda x ). $$

After simple manipulation the left side of this double inequality is reduced to $x^{1-\lambda}<(1-\lambda)x+\lambda$ while the right side to $x^{\lambda}<1-\lambda+\lambda x$, which are equivalent to the weighted arithmetic-geometric mean inequality.

To prove $A_{\lambda}< M_{\lambda}< T_{\lambda}< Q_{\lambda}$ we proceed in a similar manner by using Example 8.2. After all reduction we are in a position to show the inequality

$$(1-\lambda)x+\lambda< \sqrt{(1-\lambda)x^{2}+\lambda}, $$

which follows from the strict concavity of the real function $x\longmapsto\sqrt{x}$ on $(0,\infty)$. □

Another example of applications is given in the following result.

### Proposition 8.4

*The following inequalities hold*:

$$G_{2/3}< \mathit{SB}< A_{2/3}. $$

### Proof

First, we notice that this double inequality was already proved in the literature; see [1] for instance. Our aim here is to prove it again by using our new approach, in a fast way.

Let *k* be as in Example 8.3(ii), where we have seen that (by taking $\lambda=2/3$)

$$F_{\mathit{SB}}^{k}(x)=\frac{1}{\sqrt{|x^{2}-1|}},\qquad F_{A_{2/3}}^{k}(x)= \frac {3(2x+1)}{(x+2)^{2}\sqrt{|x^{2}-1|}},\qquad F_{G_{2/3}}^{k}(x)=\frac{x^{-4/3}(2x+1)}{ 3\sqrt{|x^{2}-1|}} $$

for all $x>0$ with $x\neq1$. With this, it is easy to verify that $F_{\mathit{SB}}^{k}(x)>F_{A_{2/3}}^{k}(x)$ for all $x>0$ with ${x\neq1}$, *i.e.* $F_{A_{2/3}}^{k}\prec F_{\mathit{SB}}^{k}$. By Proposition 8.2(ii) we deduce that $\mathit{SB}< A_{2/3}$. Now, to prove ${F_{\mathit{SB}}^{k}\prec F_{G_{2/3}}^{k}}$ we have to show that $\frac{1}{\sqrt{|x^{2}-1|}}<\frac {x^{-4/3}(2x^{2}+1)}{3\sqrt{|x^{2}-1|}}$ or equivalently (after simple reduction) $x^{4/3}<\frac{2}{3}x^{2}+\frac{1}{3}$, for all $x>0$ with $x\neq1$. We can write (by using the weighted arithmetic-geometric inequality)

$$x^{4/3}=x\cdot x^{1/3}< x \biggl(\frac{1}{3}x+ \frac{2}{3} \biggr)=\frac {1}{3}x^{2}+ \frac{2}{3}x< \frac{2}{3}x^{2}+\frac{1}{3}, $$

since the latter inequality is equivalent to $x^{2}-2x+1=(x-1)^{2}>0$ for $x\neq1$. The desired inequality follows by Proposition 8.2(ii), so completing the proof. □

## Inverse transform of $m\longmapsto m^{\sigma_{k}}$

This section displays the inverse mean-map of $m\longmapsto m^{\sigma _{k}}$. The main result here is stated as follows.

### Theorem 9.1

*Let* $k\in{\mathcal {K}}$ *and* *m* *be a homogeneous mean*. *Let* $F_{m}^{k}$ *be the* *k*-*generated of* *m*. *Assume that the function* $k_{12}:(0,\infty )\longrightarrow(0,\infty)$ *is bijective whose inverse is* $k_{12}^{-1}$. *Then the binary map* $R_{m,k}$ *defined by*

9.1 $$ R_{m,k}(a,b)=\frac{b}{k_{2}\circ k_{12}^{-1}(a/b)}F_{m}^{k} \circ k_{12}^{-1}(a/b) $$

*for all* $a,b>0$, *with* $R_{m,k}(a,a)=a$, *is a homogeneous mean*, *with the relationship* $R_{m,k}^{\sigma_{k}}=m$. *If*, *moreover*, *m* *is continuous then so is* $R_{m,k}$.

### Proof

We first show that $R_{m,k}$ is a mean. Let us set $k_{12}^{-1}(a/b)=c$ for the sake of simplicity. Since $F_{m}^{k}$ is assumed to satisfy (4.1), for all $t>0$, we have

$$\min \bigl(k_{1}(t),k_{2}(t) \bigr)\leq F_{m}^{k}(t) \leq\max \bigl(k_{1}(t),k_{2}(t) \bigr), $$

or by homogeneity

$$k_{2}(t)\min \bigl(k_{12}(t),1 \bigr)\leq F_{m}^{k}(t)\leq k_{2}(t)\max \bigl(k_{12}(t),1 \bigr). $$

In particular, taking $t=c$ we obtain

$$k_{2}(c)\min \bigl(k_{12}(c),1 \bigr)\leq F_{m}^{k}(c)\leq k_{2}(c)\max \bigl(k_{12}(c),1 \bigr), $$

or again, by virtue of $c=k_{12}^{-1}(a/b)$ *i.e.* $a/b=k_{12}(c)$,

$$\min(a/b,1)\leq\frac{1}{k_{2}\circ k_{12}^{-1}(a/b)}F_{m}^{k}\circ k_{12}^{-1}(a/b)\leq\max(a/b,1), $$

from which we deduce that $R_{m,k}$ defined by (8.2) is a mean. The homogeneity of $R_{m,k}$ is immediate.

Let us show that $R_{m,k}^{\sigma_{k}}=m$. It is very easy to verify that $R_{m,k} (k_{1}(t),k_{2}(t) )=F_{m}^{k}(t)$ for all $t>0$. Further, by (4.8) we have

$$\bigl(R_{m,k}^{\sigma_{k}}(a,b) \bigr)^{-1}= \frac{1}{k(a,b)} \int _{1}^{a/b}R_{m,k} \bigl(k_{1}(t),k_{2}(t) \bigr)\,dt=\frac{1}{k(a,b)} \int_{1}^{a/b}F_{m}^{k}(t)\,dt, $$

which with (8.2) yields the desired result.

Now, assume that *m* is continuous and prove that $R_{m,k}$ is as well. Since $R_{m,k}$ is homogeneous, it is sufficient to show that $x\longmapsto R_{m,k}(x,1)$ is continuous. By (9.1) we have

$$R_{m,k}(x,1)=\frac{1}{k_{2}\circ k_{12}^{-1}(x)}F_{m}^{k}\circ k_{12}^{-1}(x), $$

from which the continuity of $x\longmapsto R_{m,k}(x,1)$ on $(0,1)\cup (1,\infty)$ follows, since the involved functions $k_{2}$, $k^{-1}_{12}$ and $F_{m}^{k}$ are all continuous on $(0,1)\cup(1,\infty)$. For proving the continuity of $x\longmapsto R_{m,k}(x,1)$ at $x=1$ we write

$$\lim_{x\rightarrow1}R_{m,k}(x,1)=\lim_{x\rightarrow1} \frac{F_{m}^{k} (k^{-1}_{12}(x) )}{k_{2} (k^{-1}_{12}(x) )}= \lim_{y\rightarrow1}\frac{F_{m}^{k}(y)}{k_{2}(y)}, $$

since $k^{-1}_{12}$ is continuous with $k^{-1}_{12}(1)=1$. Now, by the definition of $F_{m}^{k}$ and $k_{2}$ with the (reversed) l’Hôpital rule, we have

$$ \begin{aligned} \lim_{x\rightarrow1}\frac{F_{m}^{k}(x)}{k_{2}(x)}&=\lim_{x\rightarrow1} \frac {\frac{d}{dx} (\frac{k(x,1)}{m(x,1)} )}{ \frac{d}{dx} (x^{-1}k(x,1) )}=\lim_{x\rightarrow1}\frac{\frac {k(x,1)}{m(x,1)}}{x^{-1}k(x,1)} \\ &=\lim_{x\rightarrow1} \frac{x}{m(x,1)}=\frac{1}{m(1,1)}=1=R_{m,k}(1,1), \end{aligned}$$

since *m* is continuous and $R_{m,k}(a,a)=a$ for each $a>0$. The proof of the theorem is completed. □

As consequence of the above theorem we obtain the following result which is of interest in practical purposes. For the sake of simplicity we adopt the notation $x^{1/q}=\sqrt [q]{x}$ for all $x,q>0$.

### Corollary 9.2

*Let* *k* *be defined by* (3.2) *and* *m* *be a homogeneous mean*. *Then the binary map*

$$R_{m,k}(a,b)=\sqrt[q+1]{a^{2}}\big|\sqrt[q+1]{a^{q}}- \sqrt[q+1]{b^{q}} \big|^{1/q*} F_{m}^{k} \bigl( \sqrt[q+1]{a/b} \bigr) $$

*is a homogeneous mean with* $R_{m,k}^{\sigma_{q}}=m$. *Further*, $R_{m,k}$ *is continuous if* *m* *is as well*.

### Proof

Here we have $k_{12}(t)=t^{q+1}$ and $k_{2}$ is explicitly given in Example 3.1. The desired result follows after simple computation and reduction. □

In particular, if $k(a,b)=a-b$ *i.e.* $q=1$, $1/q^{*}=0$ and $F_{m}$ denotes the generated function of *m*, then the binary map: $r_{m}(a,b)=aF_{m}(\sqrt{a/b})$ for all $a,b>0$, $a\neq b$, defines a homogeneous mean with $r_{m}^{\sigma}=m$. Further, $r_{m}$ is symmetric (resp. continuous) provided that *m* is as well. This particular situation corresponds to that developed in [18].

The above theorem tells us that starting from a homogeneous mean *m*, we can construct a lot of new homogeneous means $R_{m,k}$ whenever $k\in {\mathcal {K}}$ is given. Moreover, we have $R_{m,k}^{\sigma_{k}}=m$. Inversely, let *m* be a homogeneous mean and $k\in{\mathcal {K}}$ be fixed. Does there exist a unique homogeneous mean $r:=r_{m,k}$ such that $r^{\sigma_{k}}=m$? The following result gives a positive answer to this question. For the sake of simplicity, if ${\mathcal {M}}_{\mathrm{hc}}$ denotes the set of all homogeneous continuous means, we introduce the following notation:

9.2 $$ \Omega= \bigl\{ (m,k)\in{\mathcal {M}}_{\mathrm{hc}}\times{\mathcal {K}},F_{m}^{k} \mbox{ satisfies } (8.1) \mbox{ and } k_{12} \mbox{ is a bijection} \bigr\} . $$

### Corollary 9.3

*Let* $(m,k)\in\Omega$. *Then there exists one and only one mean* $r=r_{m,k}\in{\mathcal {M}}_{\mathrm{hc}}$ *such that* $r^{\sigma_{k}}=m$. *We then write* $r=m^{-\sigma_{k}}$.

### Proof

The existence follows from the previous theorem, since $R_{m,k}^{\sigma _{k}}=m$. The uniqueness is an immediate consequence of Proposition 5.3. Details are simple and therefore omitted here for the reader. □

Under the hypotheses of Corollary 9.3 and combining the above results, the unique mean $r\in{\mathcal {M}}_{\mathrm{hc}}$ such that $r^{\sigma _{k}}=m$ is given by $r=R_{m,k}$, where $R_{m,k}$ is defined by (9.1). We can then write $m^{-\sigma_{k}}=R_{m,k}$ and $R_{m,k}$ will be called the $\sigma_{k}$-inverse mean of *m*. We then have $(m^{-\sigma_{k}} )^{\sigma_{k}}=m$ and $(m^{\sigma_{k}} )^{-\sigma_{k}}=m$ for every $(m,k)\in\Omega$.

The following example illustrates the previous results.

### Example 9.1

(i) Following Proposition 5.1, we have $A_{\lambda}^{\sigma _{k}}=H_{1-\lambda}$. By Corollary 9.3 we then deduce that $H_{\lambda}^{-\sigma_{k}}=A_{1-\lambda}=G^{2}/H_{\lambda}$ for all $\lambda \in[0,1]$ and every $k\in{\mathcal {K}}$ satisfying the hypotheses of the previous corollary.

(ii) Example 6.2 asserts that $G_{\lambda}^{\sigma_{q}}=X_{\lambda ,q}$ for all $\lambda\in(0,1)$ and $q>0$. We can then write $X_{\lambda ,q}^{-\sigma_{q}}=G_{\lambda}$ and in particular, $\mathit{SB}^{-\sigma _{2}}=G_{1/3}$.

(iii) Theorem 7.1 asserts that $Y_{\lambda,q}^{-\sigma _{q}}=H_{\lambda}$ for every $\lambda\in(0,1)$ and $q>0$. In particular, (7.1) yields $T_{\lambda}^{-\sigma}=H_{1-\lambda}=G^{2}/A_{\lambda }$ for each $\lambda\in(0,1)$.

Other examples of interest are given in the following result.

### Theorem 9.4

*For all* $\lambda\in[0,1]$, *the following relationships hold*:

$$\begin{gathered} A_{\lambda}^{-\sigma}=\frac{G^{2}}{S_{\lambda}},\qquad G_{\lambda}^{-\sigma }= (G_{1-\lambda}S_{1-\lambda} )^{1/2}, \qquad L_{\lambda}^{-\sigma }=G_{1-\lambda}= \frac{G^{2}}{G_{\lambda}}, \\ M_{\lambda}^{-\sigma}=\frac{G^{2}}{ (A_{\lambda}S_{\lambda} )^{1/2}},\qquad Q_{\lambda}^{-\sigma}= \frac{G^{2}S_{\lambda}^{1/2}}{A_{\lambda}^{3/2}}. \end{gathered}$$

### Proof

We show, for example, the second and fourth equalities. The other ones can be proved in a similar way by using analogous tools. By definition we have

$$ \begin{aligned} G_{\lambda}^{-\sigma}(a,b)&:=aF_{G_{\lambda}} (\sqrt{a/b} )=a (a/b )^{\lambda/2-1} (1-\lambda+\lambda\sqrt{a/b} ) \\ &=a^{\lambda/2}b^{(1-\lambda)/2} \bigl((1-\lambda)\sqrt{b}+\lambda\sqrt {a} \bigr)=G_{1-\lambda}^{1/2}(a,b)S_{1-\lambda}^{1/2}(a,b), \end{aligned} $$

which is the desired result. For $M_{\lambda}^{-\sigma}$ we have

$$M_{\lambda}^{-\sigma}(a,b)=aF_{M_{\lambda}} (\sqrt{a/b} )= \frac {ab}{ ((1-\lambda)\sqrt{a}+\lambda\sqrt{b} )\sqrt{(1-\lambda )a+\lambda b}}, $$

from which the desired result follows. □

### Remark 9.1

The equalities of the previous theorem can be linked by nice and simple relationships which can be used for obtaining inequalities between the involved weighted means. For more details, see Section 11 below.

Now, we state the next result which is also of interest.

### Corollary 9.5

*Let* $m_{1},m_{2}\in{\mathcal {M}}_{\mathrm{hc}}$. *Let* $k\in{\mathcal {K}}$ *be such that* $(m_{1},k)\in\Omega$ *and* $(m_{2},k)\in\Omega$. *If* $m_{1}^{-\sigma _{k}}< m_{2}^{-\sigma_{k}}$ *then we have* $m_{1}>m_{2}$.

### Proof

This follows immediately from the definition of $m\longmapsto m^{-\sigma _{k}}$ with Proposition 5.2(iii). □

We can show again all inequalities of (7.4) by using the previous corollary. This is explained in the following example.

### Example 9.2

To show, for example, $T_{\lambda}< Q_{\lambda}$ it is sufficient to prove that $Q_{\lambda}^{-\sigma}< T_{\lambda}^{-\sigma}$ *i.e.*

$$\frac{G^{2}S_{\lambda}^{1/2}}{A_{\lambda}^{3/2}}< H_{1-\lambda}=\frac {G^{2}}{A_{\lambda}}, $$

which is reduced to $S_{\lambda}< A_{\lambda}$ well-known inequality.

We left to the reader the task for verifying the other inequalities in a similar manner.

Another example of application is given in the following.

### Example 9.3

For all $\lambda\in(0,1)$, we have $H_{\lambda}< G_{\lambda}< A_{\lambda }$. According to the construction of $X_{q,\lambda}$ and $Y_{q,\lambda }$, with Proposition 5.1 and Example 9.1, the previous double inequality is equivalent to the following one:

$$Y_{q,\lambda}^{-\sigma_{q}}< X_{q,\lambda}^{-\sigma_{q}}< H_{1-\lambda }^{-\sigma_{q}} $$

for all $q>0$. This, with Corollary 9.5 yields

$$H_{1-\lambda}< X_{q,\lambda}< Y_{q,\lambda} $$

for all $\lambda\in(0,1)$ and $q>0$.

## About the *P*-weighted mean

As already pointed out before, this section deals with the weighted mean of *P*. We will see that we can introduce more one *P*-weighted means following different point of view. We also introduce other *L*-weighted means.

First, we cannot suggest the form of $P_{\lambda}$ (directly from that of $T_{\lambda}$) as we did it for $M_{\lambda}$, *i.e.* just by replacing arctan by arcsin. This is so because the expression

$$\frac{\sqrt{\lambda(1-\lambda)}(a-b)}{A_{\lambda}(a,b)} $$

does not always belong to $[-1,1]$, for $a,b>0$. Following another tool of intuition and analyzing the generated functions associated to $P_{\lambda}$ and $M_{\lambda}$ we can suggest that

10.1 $$ \bigl(P_{\lambda}(a,b) \bigr)^{-1}= \frac{1}{b-a} \int_{1}^{a/b}\frac {t^{\lambda-1}}{(1-\lambda)t+\lambda}\,dt $$

is a weighted *P*-mean. In fact, we can easily verify that $P_{\lambda }$ satisfies all conditions of Definition 2.1. For proving $P_{1/2}=P$ we use the change of variables $\sqrt{t}=u$ while for the relation $P_{1-\lambda}(a,b)=P_{\lambda}(b,a)$ we put $t=1/u$. As in the previous study, we will give more justification for our suggestion. We first state the following result.

### Proposition 10.1

*The following relationships hold*:

$$\begin{gathered} \forall x>0,\quad F_{P_{\lambda}}(x)=\frac{x^{\lambda-1}}{(1-\lambda )x+\lambda}, \\P_{\lambda}^{-\sigma}=\frac{G^{2}}{ (G_{\lambda}S_{\lambda} )^{1/2}}. \end{gathered}$$

### Proof

The first relation immediately follows from (10.1) with Definition 8.1. For the second relation, we have (in a similar way to above)

$$P_{\lambda}^{-\sigma}(a,b)=\frac{a(a/b)^{(\lambda-1)/2}}{(1-\lambda )\sqrt{a/b}+\lambda}=\frac{a^{(1+\lambda)/2}b^{(2-\lambda )/2}}{S_{\lambda}^{1/2}(a,b)}. $$

To complete the proof it is sufficient to remark that

$$a^{(1+\lambda)/2}b^{(2-\lambda)/2}=\frac{ab}{a^{(1-\lambda)/2}b^{\lambda /2}}=\frac{G^{2}(a,b)}{G_{\lambda}^{1/2}(a,b)}. $$

 □

Now, we can state the following result giving more justification to our previous suggestion.

### Proposition 10.2

*We have* $L_{\lambda}< P_{\lambda}< A_{\lambda}$ *for all* $\lambda\in(0,1)$.

### Proof

As before, we can prove this double inequality in different ways. We present here two methods:

- By Proposition 8.2(ii), it is sufficient to show that the double inequality
  $$F_{A_{\lambda}}(x)< F_{P_{\lambda}}(x)< F_{L_{\lambda}}(x) $$
  holds for all $x>0$ with $x\neq1$. According to Example 8.1 and Proposition 10.1, we have to prove that
  $$\frac{1}{ ((1-\lambda)x+\lambda )^{2}}< \frac{x^{\lambda -1}}{(1-\lambda)x+\lambda}< x^{2(\lambda-1)} $$
  holds for all $x>0$ with $x\neq1$. The left side of this double inequality as well as its right side is reduced to $x^{1-\lambda }<(1-\lambda)x+\lambda$ which is the well-known Young (or weighted arithmetic-geometric) inequality. The desired double inequality is proved.
- Following Corollary 9.5, it is sufficient to show that
  $$A_{\lambda}^{-\sigma}< P_{\lambda}^{-\sigma}< L_{\lambda}^{-\sigma}. $$
  By Theorem 9.4 and Proposition 10.1, this is equivalent to
  $$\frac{G^{2}}{S_{\lambda}}< \frac{G^{2}}{ (G_{\lambda}S_{\lambda} )^{1/2}}< \frac{G^{2}}{G_{\lambda}}, $$
  which, in its two sides, is reduced to $G_{\lambda}< S_{\lambda}$, so finishing the proof. □

For the weighted means $T_{\lambda}$ and $M_{\lambda}$ we have seen that $T_{\lambda}=\mathit{SB}(A_{\lambda},Q_{\lambda})$ and $M_{\lambda }=\mathit{SB}(Q_{\lambda},A_{\lambda})$. Analogous relation for $P_{\lambda}$ seems to be not obvious. We then put the following as open problem.

### Problem 1

Prove or disprove that $P_{\lambda}$ defined by (10.1) satisfies $P_{\lambda}=\mathit{SB} (G_{\lambda},A_{\lambda} )$. Similar question can be posed for $L_{\lambda}=\mathit{SB} (A_{\lambda },G_{\lambda} )$.

It is worth mentioning that the weighted means $\mathit{SB} (G_{\lambda },A_{\lambda} )$ and $\mathit{SB} (A_{\lambda},G_{\lambda} )$ satisfy the following inequalities:

$$G_{\lambda}< \mathit{SB} (A_{\lambda},G_{\lambda} )< \mathit{SB} (G_{\lambda },A_{\lambda} )< A_{\lambda}. $$

Indeed, using the fact that $\mathit{SB}(x,y)<\mathit{SB}(y,x)$ for $x< y$ and $\mathit{SB}(x,y)$ is strictly increasing in *x* and *y*, we can proceed as in [1] for writing

$$G_{\lambda}=\mathit{SB} (G_{\lambda},G_{\lambda} )< \mathit{SB} (A_{\lambda },G_{\lambda} )< \mathit{SB} (G_{\lambda},A_{\lambda} ) < \mathit{SB} (A_{\lambda},A_{\lambda} )=A_{\lambda}. $$

Now, we will see that we can give other weighted means, associated to *P* and *L*, which are different from the previous ones. The previous *P*-weighted mean was constructed from an analogy of the generated functions of *P* with those of *T* and *M*. Here, we will use another point of view. Analyzing the expressions of $T_{\lambda}$ and $M_{\lambda}$, in a parallel way with those of *T* and *M*, together with the various expressions of *P* (previously mentioned in the introduction), we can suggest that

10.2 $$ P_{\lambda}(a,b)=\frac{\sqrt{\lambda(1-\lambda)}(\sqrt{a}-\sqrt {b})A_{\lambda} (\sqrt{a},\sqrt{b} )}{ \arctan\frac{\sqrt{\lambda(1-\lambda)}(\sqrt{a}-\sqrt{b})}{A_{\lambda }(\sqrt{a},\sqrt{b})}},\qquad P_{\lambda}(a,a)=a, $$

is a weighted mean of *P*. Indeed, a simple verification asserts that this $P_{\lambda}$ satisfies all conditions of Definition 2.1.

Now, we can ask what is the more reasonable *P*-weighted mean among the two previous ones. In fact, it depends on what we want to do and what we want to have. For instance, if we desire to conserve the inequalities $L_{\lambda}< P_{\lambda}< A_{\lambda}$, by analogy with $L< P< A$, then the *P*-weighted mean given by (10.1) is more convenient, since that given by (10.2) does not satisfy the previous double inequality (we omit its verification here).

Finally, for the logarithmic mean we can also give another weighted *L*-mean. This can be done if we recall that

$$L(a,b)=\frac{a-b}{\ln a-\ln b}=\frac{a-b}{2\operatorname{arctanh}\frac{a-b}{a+b}} $$

for all $a,b>0$, $a\neq b$. Now, the idea is clear and we can proceed as previously. We leave to the reader the task for deducing another weighted *L*-mean and to compare it with the previous one.

## Inequalities involving the previous weighted means

As pointed out before, we present here some inequalities involving three means among the previous weighted means. For this purpose, we need a list of theoretical results which we will state in what follows. We begin by the first proposition.

### Proposition 11.1

*The following relationships hold true*:

$$\begin{gathered} A_{\lambda}^{-\sigma}T_{\lambda}^{-\sigma}= \bigl(M_{\lambda}^{-\sigma } \bigr)^{2},\qquad M_{\lambda}^{-\sigma}Q_{\lambda}^{-\sigma}= \bigl(T_{\lambda}^{-\sigma } \bigr)^{2},\\ L_{\lambda}^{-\sigma}A_{\lambda}^{-\sigma}= \bigl(P_{\lambda }^{-\sigma} \bigr)^{2},\qquad A_{\lambda}^{-\sigma}Q_{\lambda}^{-\sigma}=M_{\lambda}^{-\sigma }T_{\lambda}^{-\sigma}. \end{gathered}$$

### Proof

This follows immediately from Theorem 9.4 and Proposition 10.1. Details are simple and therefore omitted here. □

Now, we state the following result.

### Theorem 11.2

*Let* $k\in{\mathcal {K}}$ *be fixed*. *Then the map* $m\longmapsto m^{\sigma _{k}}$ *enjoys the following properties*:

(i) *Point*-*wise convexity*: *for all* $\alpha\in(0,1)$ *and any means* $m_{1}$ *and* $m_{2}$ *we have*
  $$\bigl((1-\alpha)m_{1}+\alpha m_{2} \bigr)^{\sigma_{k}} \leq(1-\alpha)m_{1}^{\sigma _{k}}+\alpha m_{2}^{\sigma_{k}}. $$
  *If*, *moreover*, $m_{1}\neq m_{2}$ *are comparable the previous mean inequality becomes strict*.
(ii) *Point*-*wise geometric strict concavity*: *for all* $\alpha\in(0,1)$ *and any means* $m_{1}\neq m_{2}$ *one has*
  11.1 $$ \bigl(m_{1}^{1-\alpha}m_{2}^{\alpha} \bigr)^{\sigma_{k}}> \bigl(m_{1}^{\sigma_{k}} \bigr)^{1-\alpha} \bigl(m_{2}^{\sigma_{k}} \bigr)^{\alpha}. $$

### Proof

It is similar to those of Theorem 4.2 and Theorem 4.4 of [18], pages 96-97, with some precautions. Details are omitted here to the aim to not lengthen the present paper. □

In applications, the following corollary is of interest.

### Corollary 11.3

*Let* $m_{1}$, $m_{2}$, *m* *be three means*, *with* $m_{1}\neq m_{2}$. *Let* $k\in{\mathcal {K}}$ *be such that* $(m_{1},k)\in\Omega$, $(m_{2},k)\in\Omega$ *and* $(m,k)\in \Omega$, *where* Ω *was defined by* (9.2). *Assume that*

11.2 $$ m^{-\sigma_{k}}\leq \bigl(m_{1}^{-\sigma_{k}} \bigr)^{1-\alpha} \bigl(m_{2}^{-\sigma _{k}} \bigr)^{\alpha} $$

*for some* $\alpha\in(0,1)$. *Then we have*

$$m_{1}^{1-\alpha}m_{2}^{\alpha}< m. $$

### Proof

From (11.2), with Proposition 5.2(ii), we obtain

$$\bigl( \bigl(m_{1}^{-\sigma_{k}} \bigr)^{1-\alpha} \bigl(m_{2}^{-\sigma_{k}} \bigr)^{\alpha} \bigr)^{\sigma_{k}} \leq \bigl(m^{-\sigma_{k}} \bigr)^{\sigma_{k}}=m. $$

This, with (11.1), immediately yields the desired mean inequality. □

Now, we will illustrate the previous statements with the following example.

### Example 11.1

(i) The relationship $A_{\lambda}^{-\sigma}T_{\lambda}^{-\sigma}= (M_{\lambda}^{-\sigma} )^{2}$ of Proposition 11.1 can be written as

$$M_{\lambda}^{-\sigma}= \bigl(A_{\lambda}^{-\sigma} \bigr)^{1/2} \bigl(T_{\lambda}^{-\sigma} \bigr)^{1/2}, $$

which is (11.2) as equality, with $\alpha=1/2$, $k(a,b)=a-b$ and $m=M_{\lambda}$, $m_{1}=A_{\lambda}$, $m_{2}=T_{\lambda}$. We immediately deduce, by Corollary 11.3, that $M_{\lambda}^{2}>A_{\lambda}T_{\lambda}$ for any $\lambda\in(0,1)$.

(ii) By similar arguments, we show that the two mean inequalities

$$T_{\lambda}^{2}>M_{\lambda}Q_{\lambda} \quad\mbox{and}\quad P_{\lambda }^{2}>A_{\lambda}L_{\lambda} $$

hold for any $\lambda\in(0,1)$.

(iii) We leave to the reader the routine task for obtaining more mean inequalities in a similar way to previously.

We end this paper by stating the following remark.

### Remark 11.1

The mean inequalities obtained in Example 11.1, for $\lambda\in (0,1)$, justify again that $L_{\lambda}$, $M_{\lambda}$, $T_{\lambda}$ and $P_{\lambda}$ are reasonable weighted means of *L*, *M*, *T* and *P*, respectively. This is so because, for $\lambda=1/2$, they yield the known mean inequalities $M^{2}>AT$, $T^{2}>MQ$ and $P^{2}>AL$, already proved in [2].
